# Plasmacytoid Dendritic Cells: Neglected Regulators of the Immune Response to *Staphylococcus aureus*

**DOI:** 10.3389/fimmu.2014.00238

**Published:** 2014-05-23

**Authors:** Isabelle Bekeredjian-Ding, Johann Greil, Sandra Ammann, Marijo Parcina

**Affiliations:** ^1^Institute for Microbiology, Immunology and Parasitology, University Hospital Bonn, Bonn, Germany; ^2^Department of Pediatrics, University Hospital Heidelberg, Heidelberg, Germany; ^3^Department of Infectious Diseases, Medical Microbiology and Hygiene, University Hospital Heidelberg, Heidelberg, Germany

**Keywords:** pDC, *Staphylococcus aureus*, type I interferons, immune complexes, bacteria, tolerance, autoimmunity, allergy

## Abstract

Plasmacytoid dendritic cells (pDC) are a rare subset of leukocytes equipped with Fcγ and Fcε receptors, which exert contrary effects on sensing of microbial nucleic acids by endosomal Toll-like receptors. In this article, we explain how pDC contribute to the immune response to *Staphylococcus aureus*. Under normal circumstances the pDC participates in the memory response to the pathogen: pDC activation is initiated by uptake of staphylococcal immune complexes with IgG or IgE. However, protein A-expressing *S. aureus* strains additionally trigger pDC activation in the absence of immunoglobulin. In this context, staphylococci exploit the pDC to induce antigen-independent differentiation of IL-10 producing plasmablasts, an elegant means to propagate immune evasion. We further discuss the role of type I interferons in infection with *S. aureus* and the implications of these findings for the development of immune based therapies and vaccination.

## Introduction

Plasmacytoid dendritic cells (pDC) constitute a rare, but ubiquitously present leukocyte subset. They were originally described as professional interferon-producing cells (IPC) (1, 2), because of their capacity to secrete high concentrations of type I interferons (IFN-I) (mainly IFN-α) that can reach systemic activity [reviewed in Ref. (3, 4)].

Numerous studies elucidate pDC function in different types of infection, autoimmune disease entities and in maintenance of tolerance [reviewed in Ref. (5)]. Most importantly, they have made us aware of the fact that there are pDC functions beyond IFN secretion. Despite their versatility only few studies described pDC activation and addressed its role in the host response to *Staphylococcus aureus* (6–12). Thus, their function in the daily combat with colonizing and infecting *S. aureus* strains remains to be investigated. In this article, we summarize the available findings and discuss the potential implications for immune based therapies and vaccine design.

### Well-equipped viral sensors

Type I interferons are essential for antiviral immune defense. Early studies postulated that pDC represent specialized viral sensors in first line innate immune defense against viruses [reviewed in Ref. (13)]. Identification of Toll-like receptors (TLR)-7 and -9 as the major triggers for pDC activation fostered this concept because RNA viruses such as influenza, RSV, VSV and HIV engage TLR7, and DNA viruses, i.e., HSV and EBV, activate pDC in a TLR9-dependent manner. Nevertheless, under defined circumstances cytosolic, RIG-I like-receptor-dependent recognition also occurs (8, 14–16).

Subsequent studies elucidated pDC-mediated effects on the adaptive immune response. The major findings implied that pDC-derived IFN-α supports Th1 responses [reviewed in Ref. (17)] and enhances the formation of antibody secreting cells in response to virus (18).

**Figure 1:**
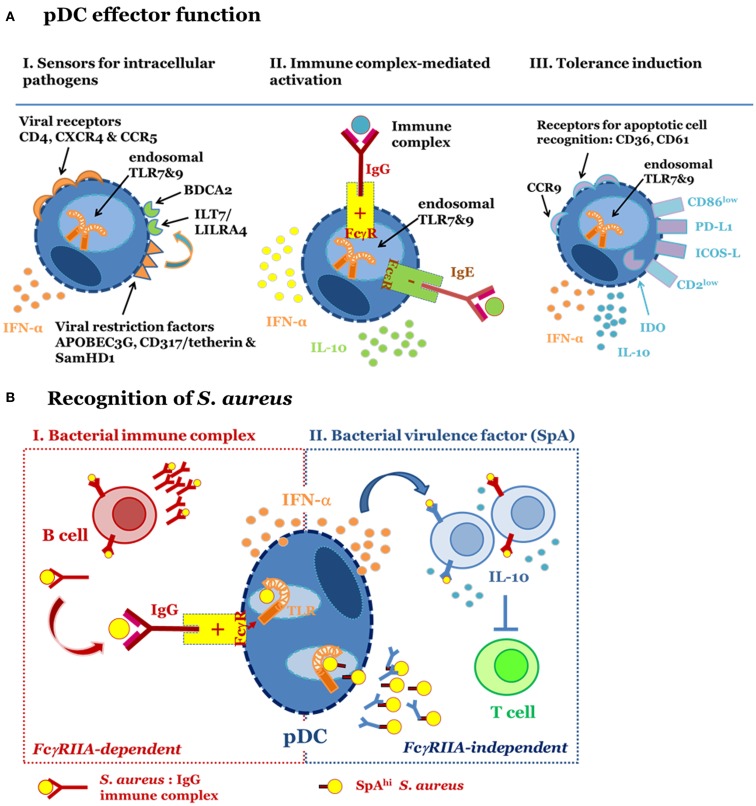
**Plasmacytoid dendritic cells function in different immune contexts**. **(A)** pDC fulfill different effector functions. I. Sensors for intracellular pathogens. pDC are target cells for viral infection because they express cell surface receptors utilized for virus entry, i.e., HIV permissive receptors CD4, CXCR4, and CCR5. Endosomal TLR7 and TLR9 mediate recognition of RNA and DNA viruses, respectively. IFN-I synthesis and viral restriction factors counteract viral infection and intracellular replication. Surface receptors such as ILT7 and BDCA2 regulate pDC function. II. Immune complex-mediated activation. pDC express FcγRIIA and FcεRI. These receptors are engaged by immune complexes consisting of IgG and bacteria, viruses or endogenous nucleic acids or IgE-bound allergens. Binding of FcγRIIA promotes access of IgG-complexed nucleic acids to endosomal TLR and enables pDC-derived IFN-α release. FcεRI ligation inhibits TLR9-induced IFN-α secretion and induces IL-10 production and Th2 polarization. III. Tolerance induction. pDC promote tolerance via induction of Treg. Tolerogenic function has been associated with high PDL-1 and low CD86 expression, release of IL-10, expression of CCR9, IDO, ICOS-L, and low CD2. Expression of CD36 and CD61 among other surface receptors enables apoptotic cell recognition. **(B)** *S. aureus*-induced pDC activation occurs in an FcγRIIA-dependent (I) and -independent (II) fashion. I (left): pDC are activated by bacterial immune complexes (IC) with anti-staphylococcal IgG. This activation requires prior generation of IgG and therefore forms part of a secondary immune response. IC-mediated engagement of the FcγRIIA promotes access of staphylococcal nucleic acids to endosomal TLR7/9, which induces secretion of IFN-α. II (right): *S. aureus* strains expressing high levels of protein A (SpA) stimulate pDC in the absence of IgG and IC formation. This virulence factor-dependent mechanism for pDC activation triggers release of IFN-α via activation of endosomal TLR7/9. Activated pDC support SpA-dependent B cell expansion and differentiation into IL-10 secreting plasmablasts. B cell-derived IL-10 production, a hallmark of regulatory B cell function, prevents T cell responses by inhibiting antigen presentation by dendritic cells and macrophages.

The relevance of pDC and IFN-I in antiviral immune defense is strongly supported by the multitude of viral immune escape mechanisms interfering with the induction of IFN-I or its direct effect on intracellular viral replication. Depletion of pDC in *in vivo* infection models revealed that pDC contribute to virus clearance and constrain inflammation during infection (19–21). Concomitant expression of HIV permissive receptors, i.e., CD4, CXCR4, CCR5 [reviewed in Ref. (22, 23)], and viral restriction factors (Figure 1A, left panel), i.e., APOBEG3G (24), CD317/tetherin/BST2 (25), ILT7 (26), and SamHD1 turns pDC into “viral traps,” e.g., important target cells for viral infection albeit well-equipped and highly specialized on intracellular antiviral defense (Figure 1A, left panel).

### Perpetuators of autoimmune disease and allergy

Due to their highly efficient IFN-α producing capacity it was obvious that pDC might be involved in autoimmune disease. In this disease context, pDC were shown to be activated by immune complexes (IC) consisting of autoantibodies binding endogenous chromatin or RNA and activating TLR9 or TLR7, respectively [reviewed in Ref. (27–30)]. Uptake of IC was mediated via FcγRIIA (CD32A), a positive regulatory receptor for IgG (Figure 1A, middle panel). IC-induced pDC-derived IFN-α is thought to account for elevated IFN-I levels in patients with systemic lupus erythematosus, Behcet’s disease, and Sjögren’s syndrome (31–33). The ubiquitous presence of pDC facilitates systemic effects of IFN-α. This cytokine contributes to the development and severity of disease by enhancing autoantibody production and driving inflammation [reviewed in Ref. (34)].

Similarly to autoantigens allergens complexed with IgE can activate pDC. However, ligation of the FcεRI expressed on pDC has quite distinct effects from those induced via FcγRIIA activation: FcεRI aggregation has negative regulatory impact on influenza virus and TLR9-mediated release of IFN-I from pDC (35–37). On the contrary, aggregation of the FcεRI promotes IL-10 secretion and shifts the T cell response toward an allergy related-Th2 response (35), an effect supported by the absence of IFN-I. A correlation of serum IgE with an increased pDC/mDC ratio and a Th2 response underlines the clinical relevance of these findings (35, 36, 38).

Taken together, these examples demonstrate that pDC function varies depending on the disease entity. They highlight the dominant role of IC recognition in pDC activation and the opposite effects of differentially composed IC on pDC function.

### Mediators of tolerance

More recently, several groups have drawn our attention to the tolerogenic properties of pDC [reviewed in Ref. (5, 39)]. This includes immune regulatory function in graft-versus-host disease (40–42), cancer (26, 43), infection (44–46), autoimmune disease (47–49), and tolerance to oral antigens (50). Furthermore, pDC recognize membrane microparticles released from apoptotic cells (16, 51) and play a crucial role in the promotion of tolerance upon apoptotic cell recognition (52).

To date the tolerogenic function has been attributed to the induction of T regulatory cells (44, 53–59). Release of pDC-derived IL-10 and expression of IDO, PD-L1, and ICOS-L represent the mainstay for Treg induction (51, 54, 55, 57, 60–67) (Figure 1A, right panel). Furthermore, pDC effector function is subject to strong regulation by the cellular environment (43, 44, 60, 68–70): while TGFβ exposure promotes pDC-derived secretion of high levels of IL-6 and development of Th17 cells (71), soluble factors released from macrophages exposed to apoptotic cells prime pDC for Treg induction (52). Furthermore, TNF, ROS, IL-10, PGE_2_, and TGFβ in Peyer’s patches, tumor cells, or adjacent monocytes modulate cytokine secretion patterns and decrease IFN-α secretion levels in pDC (68, 72–75), concomitantly reducing their Th1 induction potential [reviewed in Ref. (76)].

In this context, it is further important to note that IFN-I do not exclusively act as amplifiers of inflammatory processes but also participate in Treg induction and suppression of T cells, B cells, and innate immune cells (17, 77–89). Depending on the cellular context pDC-derived IFN-I may, thus, play a role in terminating or perpetuating the adaptive immune response.

## Recognition of *Staphylococcus aureus*

### Sensing of *Staphylococcus aureus*

For a long time, pDC were considered specialized mediators of antiviral defense and only few reports addressed their ability to respond to fungi and bacteria [reviewed in Ref. (90)]. These studies focused on intracellular bacteria (91–96). Induction of IFN-I by extracellular bacteria, was only observed with *Escherichia coli* and, most importantly, *S. aureus* (9–11, 97, 98). The cellular source and the molecular mechanism triggering IFN-I release remained ill-defined.

This prompted us to investigate how *S. aureus* induces IFN-I secretion. Our data identified pDC as the major source for *S. aureus*-induced release of IFN-α from human peripheral blood leukocytes (7, 8). This finding confirmed earlier reports on IFN-α secretion in response to stimulation with *S. aureus* in a murine DC cell line (9) and initial descriptions of *S. aureus*-responsive IPCs (10, 11). Michea et al. further demonstrated uptake of *S. aureus* into pDC and secretion of IFN-α, TNF, and IL-6 (6). We further detected pDC-derived IL-1β secretion and low levels of IL-10 in response to *S. aureus* (7).

As with viruses any inhibition of endosomal maturation and of TLR7 and TLR9 activation interfered with *S. aureus*-mediated IFN-α secretion (7, 8) and staphylococcal DNA and RNA induced pDC-derived IFN-α production (8). Furthermore, stimulation with viable staphylococci was superior to that induced by non-replicating (heat- or UV-killed) cells, a finding attributed to a higher content in microbial nucleic acids and the presence of vita-PAMPs (99). Thus, pDC-derived IFN-α secretion triggered by *S. aureus* is driven by intracellular nucleic acid recognition.

Moreover, soluble factors released from tonsillar epithelial cells exposed to *S. aureus* inhibit pDC-derived cytokine production (6) and release of IFN-α in response to *S. aureus* was reduced in pDC isolated from patients suffering of hepatitis B virus infection (100). Taken together, these data highlight the importance of the cellular environment in the regulation of pDC function in *S. aureus* infection.

### pDC activation forms part of the memory response to *S. aureus*

**Figure 2:**
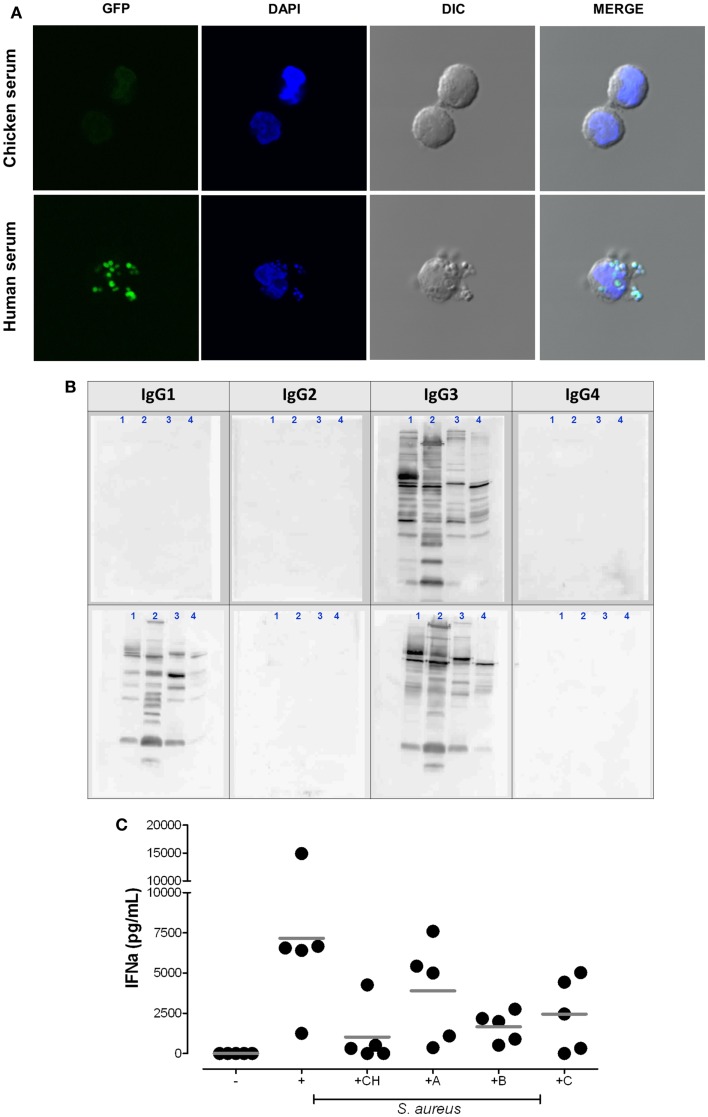
**Plasmacytoid dendritic cell activation by *S*. aureus requires the uptake of bacterial immune complexes. (A)** Cellular uptake of *S. aureus* depends on the presence of mammalian IgG. pDC were purified with anti-BDCA-4 microbeads and stimulated for 2 h at 37°C with heat-inactivated (1 h 65°C) GFP-expressing *S. aureus* strain SA113 WT (green) in medium supplemented with 5% chicken serum (upper panel) or 5% human serum (lower panel) at 37°C. DAPI (blue) was used as nuclear counterstain. Cells were visualized by confocal microscopy. **(B,C)** IgG subclass requirements for bacterial immune complex formation. **(B)** Western blot analysis of IgG subclass distribution in two patients with selective IgG deficiency. Bacterial protein lysates from USA300 (1), Newman (2), Wood46 (3), and SA113 Δ*spa* (4) were loaded on SDS-PAGE. Western blot analysis visualizes the IgG subclasses present in serum from patient 1 (IgG_3_ only) and patient 2 (IgG_1_ and IgG_3_ only, selective IgG_2/4_-deficiency). Membranes were incubated with serum followed by biotinylated anti-human IgG subclass antibodies and streptavidin-HRP as described in Ref. (7). **(C)** pDC medium was supplemented with 5% chicken serum (CH), autologous serum [healthy adult donor **(A)**], or serum from patient 1 **(B)** or patient 2 **(C)**. IFN-α release from pDC was quantified in the supernatants after 24 h stimulation. The graph summarizes the results obtained in five experiments with independent pDC donors.

Interestingly, the available reports indicated that only *S. aureus* triggers IFN-α, while other extracellular bacteria including coagulase negative staphylococci or *Streptococcus pyogenes* lack the ability to activate pDC (8, 90, 101). Figure 2A elucidates a key finding: rapid uptake of *S. aureus* into pDC is mediated by human serum containing IgG, but is absent if pDC are stimulated in serum lacking Ig with binding affinity to human FcγRs (chicken serum). Furthermore, neutralization of the FcγRIIA on pDC or absence of serum IgG blocks bacterial endocytosis and, thus, prevents access of bacterial nucleic acid to endosomal TLRs and subsequent IFN-α induction (8).

Altogether, it became clear that *S. aureus*-induced release of pDC-derived IFN-α depends on the formation of bacterial IC as propagated in autoimmune disease (8) (Figure 1B, left panel). These complexes contain anti-bacterial IgG that engages the FcγRIIA and promotes uptake of bacteria. Consequently, pDC activation occurs in an antigen-specific manner, e.g., *S. aureus*-specific IgG was required for the induction of IFN-α release. This was demonstrated by the absence of IFN-α induction when IC formation was prevented by use of a *S. aureus* strain deficient in the antigen targeted by the antibody (8).

The existence of strain- or species-specific serum IgG antibody is, thus, a key determinant in IC-induced pDC activation (8). Due to continuous exposure of humans (and animals) to *S. aureus* sera normally contain IgG specific for *S. aureus*, which explains their predisposition to respond to *S. aureus* with IFN-α production. This observation further explains why other extracellular bacteria lack IFN induction potential: they are less likely to mount an IgG memory response because humans are either rarely exposed to the bacterial species (for example *S. carnosus* found in meat) or they do not trespass the skin and/or mucosal barriers and, thus, remain secluded from the blood and lymphoid compartments and the necessity to mount an IgG response (for example *S. epidermidis*, resident of the skin). As a consequence, only individual donors display a pDC response to coagulase negative staphylococci while donor pDC normally respond to *S. aureus*.

To further elucidate the IC composition we retrieved to sera from children with selective IgG deficiencies. The data obtained showed that the presence of anti-*S. aureus* IgG_1_ and/or IgG_3_ (Figure 2B) is sufficient for the induction of pDC-derived IFN-α (Figure 2C). Thus, the presence of IgG_2_ and IgG_4_ does not represent a prerequisite for pDC activation.

Taken together, the available findings imply that prior generation of *S. aureus*-specific IgG_1/3_ is a mainstay for pDC activation. We, therefore, previously proposed that pDC activation is part of the secondary immune response to the microbe (Figure 1B, left panel). Notably, FcγRII-dependent induction of IFN-I or activation of pDC was also reported for viruses that do not actively infect pDC (102–107). We, therefore, concluded that IC-mediated pDC activation represents the physiological immune response to infection.

### Exploiting pDC for immune evasion

It is well acknowledged that *S. aureus* induces T cell-independent polyclonal B cell activation by targeting of V_H_3+ B cell receptors with the immunoglobulin-binding protein “protein A” (108). Similarly to B cells, staphylococcal protein A also induces pDC-derived IFN-α, thus circumventing the requirement for *S. aureus*-specific IgG and IC formation (7). These findings incremented earlier reports that proposed a role for protein A in IPC activation (10, 12). This alternative, albeit weaker, mechanism for pDC activation is, again, mediated by bacterial nucleic acid recognition because interference with endosomal TLR signaling abrogated pDC-derived IFN-α secretion (7). However, the exact cellular mechanisms mediating protein A-dependent uptake of nucleic acids into pDC remain to be clarified. Based on the results shown in Figure 2A, it can be speculated that IC/FcγRIIA-mediated pDC activation promotes rapid uptake of whole bacteria while protein A-induced activation could be slower, less efficient, and might be mediated by protein A-bearing peptidoglycan particles complexed with nucleic acids.

Nevertheless, this form of pDC activation is noteworthy, because it depends on a bacterial virulence factor. It, therefore, resembles viral pDC infection and might contribute to disease pathology rather than immune defense (Figure 1B, right panel).

Interestingly, protein A-dependent cytokine induction mainly affected pDC-derived IFN-α and, additionally, monocyte-derived IL-10 (7). Well in line with a report on the immune recognition of *S. aureus* in monocytes (109) we did not detect IFN-α secretion in monocytes. IL-1β levels in monocytes were lower than those in pDC and did not correlate with protein A expression levels (7), a finding well compatible with its induction by a completely distinct set of bacterial and host factors, i.e., hemolysins, peptidoglycan degradation, ATP, ASC, cryopyrin, and Nlrp3 inflammasome activation (110–114).

What is more, activation of B cells was not only paralleled by *S. aureus*-induced activation of pDC but presence of pDC was a requirement for B cell proliferation and differentiation in response to stimulation with *S. aureus* (7). Although, synergy of B cells and pDC has been described in other contexts (115–119), it is often viewed as an effect of IFN-I on B cells. However, IFN-α inhibited *S. aureus*-induced B cell proliferation as in Ref. (120–122) and pDC function cannot be substituted by IFN-α or pDC supernatants indicating that cell-to-cell contact is required (7, 119).

Altogether, this study highlighted the fundamental role of pDC in promoting polyclonal B cell expansion and mediating B cell-derived IL-10 production in an IFN-I independent manner (7). Based on these results, we propose that *S. aureus* exploits pDC to trigger polyclonal expansion of “regulatory B cells” and suppression of anti-bacterial T cell responses by B cell-derived IL-10 (123) (Figure 1B, right panel).

### Role of type I interferon in *S. aureus* infection

In contrast to virus infection, the role of IFN-α/β production in innate defense against bacterial pathogens is less clear and often paradox. While IFNαR signaling in some cases is required for bacterial clearance (124, 125), it supports hyperinflammation and increases disease severity in others (126). Albeit earlier studies reported release of IFN-α from human PBL in response to *S. aureus* (9–12, 127) a more recent study demonstrated that lysozyme resistance of *S. aureus* prevents IFN-β induction (109). Degradation-sensitive mutants induced higher levels of IFN-β, which mediated bacterial clearance *in vitro* and *in vivo* at the cost of increased inflammation and necrosis *in vivo*. IFN-β induction in monocytes was MyD88-dependent but independent of TLR, thus, arguing for a role of IL-1β or IL-18 and the inflammasome in this process. Alternatively, in the murine model TLR13-mediated sensing of staphylococcal RNA might play a contributory role in *S. aureus* recognition (128).

In other *S. aureus* infection models lack of IFNαR or TLR9 protects mice against *S. aureus* pneumonia (129, 130), an observation attributed to reduced inflammation. On the contrary, IFN-I release in LCMV infection decreases IL-12 and IFNγ production in response to *S. aureus* and predisposes mice for superinfection with *S. aureus* by inducing neutropenia (131, 132). Nevertheless, CpG ODN-mediated induction of IFN-α in pDC was protective against *S. aureus* pneumonia in a hemorrhagic shock model (133) and IFNα mediated resistance of host cells against *S. aureus* alpha toxin (134). In views of these conflicting results, we conclude that the role of type I interferon in the immune response to *S. aureus* is still ill-defined.

## Implications and Outlook

Our summary of pDC function in the immune response illustrated that pDC function depends on the cellular environment and the disease entity (Figure 1A). pDC and IFN-I have partially overlapping functions and exert numerous, partially antagonistic regulatory functions within the immune response. The complexity of this cooperation is hard to tackle.

In the context of *S. aureus* infection, we have proposed that pDC serve two purposes (Figure 1B): firstly (Figure 1B, left panel), pDC respond to uptake of IgG-containing bacterial IC with production of IFN-α. Thus, pDC activation forms part of the secondary immune response to the pathogen. Here, pDC might enhance the efficacy of memory T and B cell responses and possibly limit the priming of naïve lymphocytes. Furthermore, preferential formation of anti-staphylococcal IgG_4_, an IgG subclass with low affinity to the human FcγRIIA (7, 135–137), could contribute to avoidance of pDC activation by IgG_1_ and IgG_3_-bearing IC (Figures 2B,C), thereby weakening the memory response.

Notably, to date no study has addressed the role of IC consisting of bacteria and IgE in pDC activation. Since it is well-known that there is significant production of IgE against *S. aureus* and its toxins in nasal polyposis, asthma, and atopic dermatitis pDC might serve as important promoters of Th2-based immune responses and staphylococcal colonization in these diseases (138–143).

Secondly, *S. aureus* induces a virulence factor-driven activation of pDC (Figure 1B, right panel). Our data suggest that pathogen-driven activation of pDC enhances polyclonal B cell expansion and the development of suppressory B cells that secrete IL-10. Indeed, depletion of B cells from PBMC increases IFNγ production in response to *S. aureus* (Bekeredjian-Ding, unpublished observation). Activation of pDC by *S. aureus* might additionally promote the formation of Tregs (144), which develop in an IFN-I-dependent and -independent manner (17, 39, 145). Nevertheless, pDC-derived IFN-α is likely to enhance proinflammatory effects exerted by aberrant IC formed by Ig binding to protein A and deposited in the tissues (146), superantigen-mediated T cell activation (147), or invasion of epithelial cells (129). It is further unknown whether and how pDC contribute to *S. aureus* protein A-mediated expansion of B cells and autoantibody production in Wegener’s granulomatosis (148).

Taken together, the available studies illustrate that pDC (and IFN-I) fulfill partially unknown and conflicting roles in anti-bacterial defense and maintenance of tolerance. It is further unclear whether the active role of pDC in the memory response to *S. aureus* is to limit or to propagate the ongoing adaptive immune response. In views of the inflammatory potential of IFN-I future studies will need to evaluate whether therapeutical interference with pDC activation or IFN-I limits disease severity at a late stage of disease. However, suppression of IFN-I production in an early phase of infection might negatively affect immune recognition and favor intracellular persistence and development of chronic infections.

To date, it is further hard to foresee whether the induction of IFN-I by TLR7/9-based vaccine adjuvants will be beneficial by augmenting the preexisting memory response to *S. aureus* or converting its effector function to increased protection. It is also within the range of possibilities that these TLR agonists support the tolerogenic response exerted toward a commensal pathogen well-known to the host immune system.

## Conflict of Interest Statement

The authors declare that the research was conducted in the absence of any commercial or financial relationships that could be construed as a potential conflict of interest.
